# Role of Atomicity and Interface on InO_*x*_-TiO_2_ Composites: Thermo-Photo Valorization of CO_2_

**DOI:** 10.1021/acsami.4c04803

**Published:** 2024-06-18

**Authors:** Rocío Sayago-Carro, Irene Barba-Nieto, Uriel Caudillo-Flores, Álvaro Tolosana-Moranchel, José A. Rodríguez, Marcos Fernández-García, Anna Kubacka

**Affiliations:** † Instituto de Catálisis y Petroleoquímica, CSIC, C/Marie Curie 2, Madrid 28049, Spain; ‡ Chemistry Division, Brookhaven National Laboratory, Upton, New York 11973, United States; § Centro de Nanociencias y Nanotecnología, Universidad Nacional Autónoma de México, Ensenada 22800, México; ∥ Department of Chemistry, Stony Brook University, Stony Brook, New York 11794, United States

**Keywords:** CO_2_ elimination and valorization, thermo-photo reaction, composite catalysts, interface and size effects, indium oxide, titania

## Abstract

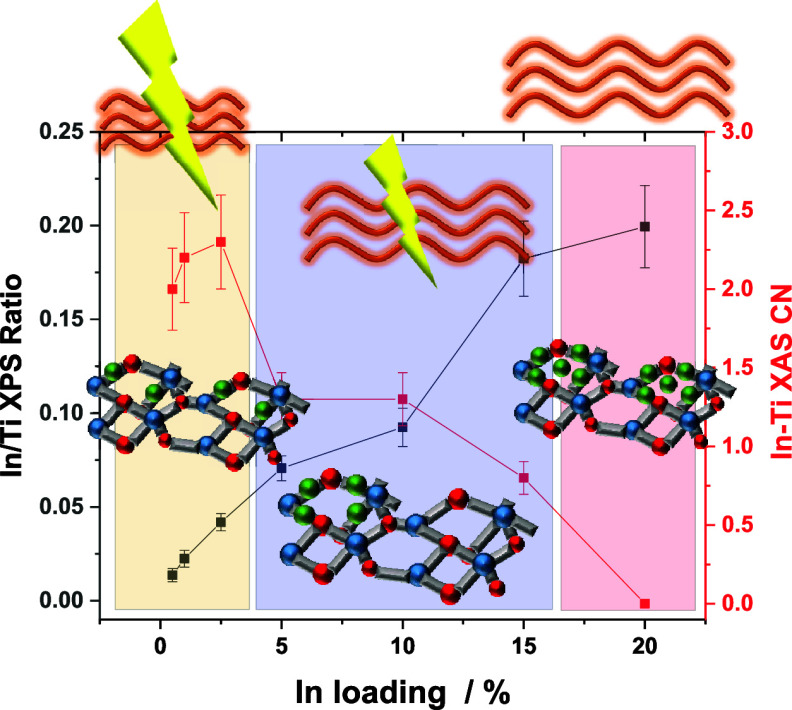

The synthesis, physicochemical, and functional properties of composite solids resulting from the surface spread of oxidized indium species onto nanoplatelets of anatase were investigated. Both the size and the interaction between the indium- and titanium-containing components control the functional properties. In the reduction of CO_2_ to CO, the best samples have an indium content between ca. 2 and 5 mol % and showed an excess rate over the photo and thermo-alone processes above 33% and an energy efficiency of 1.3%. Subnanometric (monomeric and dimeric) indium species present relatively weak thermal catalytic response but strong thermo-photo promotion of the activity. A gradual change in functional properties was observed with the growth of the indium content of the solids, leading to a progressive increase of thermal activity but lower thermo-photo promotion. The study provides a well-defined structure–activity relationship rationalizing the dual thermo-photo properties of the catalysts and establishes a guide for the development of highly active and stable composite solids for the elimination and valorization of CO_2_.

## 1 Introduction

The CO_2_ global emissions taking place from the beginning of the Industrial Revolution are continuously accelerated as a consequence of growing industrial activities as well as those derived from transportation, heating, and other needs of the increasing human population. As is well known, the CO_2_ molecule is considered the main greenhouse gas.^1,2^ Currently, it is estimated that its concentration in the atmosphere is around 420 ppm, an amount that seriously affects human health and the environment.^3^ The harmful consequences derived from CO_2_ and the so-called global warming effect on the environment have been pointed out for several decades and are now well established. Although complex, the influence of the presence of this molecule in the atmosphere in the handling of sun radiation by Earth, its effects in the acidification of oceans, the melting of glaciers, and other phenomena trigger a chain of events that would end up in unintended climate change and disruption of global ecosystems.^4,5^ Intensive CO_2_ emissions are thus attempted to be tackled down with the development of numerous technologies derived from the use of renewable energy sources and reactants, CO_2_ capture, and its conversion and valorization.^6−8^ Reduction processes have shown that they can transform carbon dioxide into value-added products (e.g., synthesis gas, methanol, hydrocarbons including light olefins, etc.), which can help essentially solve global warming.^9^ Catalysis plays a central role in many of these technologies, particularly in the efficient conversion and valorization of the molecule.^10,11^ Any chemical transformation of the CO_2_ molecule faces the fundamental problem of high stability of the C–O bond. Therefore, most conversion procedures require high energy input and suitable catalysts, which usually present limited activity and low stability. This makes necessary to research new catalysts that would meet these challenging requirements.^12^ Nevertheless, catalysis allows the transformation and simultaneous valorization of the molecule, a competitive advantage over other technologies, with a capital contribution to the circular economy and green chemistry.^10,13^

Most of the current solutions to catalytically transform the CO_2_ molecule are based on conventional, thermal processes, although electro- and photobased processes have emerged in relatively recent dates. All of them face significant challenges derived from stability under long-term operation and limited product selectivity, as well as relatively low efficiency and high cost. The latter mostly comes from the high temperature required in thermal based solutions, the energy-intensive use inherent to the electrochemical processes, or the low quantum efficiency of photobased ones.^10,11^ Dual energy catalytic solutions using binary energy sources have been explored to mitigate these inconveniences. The combination of light and heat sources allows for exploring the so-called thermo-photo or photothermal effect. Here, we use the first term to highlight possible advantages with respect to classical thermal operation conditions. The thermophotosolution can thus braid positive aspects of both thermal and light-related processes. In particular, it can reduce the energy consumption by, for example, a decrease of the temperature of operation and/or the concomitant increasing quantum efficiency of photon usage.^14−18^ This is based on the fact that the potential energy surface where the chemical reaction takes place is different in thermal and photo based catalytic processes. First, the excited states reached by thermal and photon process are different and based on different (roto-vibrational, electronic) mechanisms, but also due to the fact that dominant adiabatic (thermal) or nonadiabatic (light) relaxations of the excited intermediates would take place.^19,20^ Thus, a key point in combining two energy sources is that the new potential energy governing the reaction can lead to a benefit directly derived from an improved efficiency. This condition is obviously required and it is at the same time proven by a synergistic use of the energy sources.^14,16,17,20,21^ Nonetheless, the challenges associated with this task are huge. To point out the significance of the energetic challenge for the thermo-photo valorization of CO_2_, we note that, to the best of our knowledge, the top reported energy efficiency is well below 1%.^22^ In parallel, any new catalytic process should solve the other fundamental problem of CO_2_ valorization, resulting from the poor selectivity usually achieved.^14,16,17^

The thermo-photo catalytic valorization of the CO_2_ molecule takes place using reduction pathways leading to several industrial-oriented products and nowadays appears as a rather hot topic.^16,23−25^ As discussed above, the successful implementation of the thermophotovalorization of CO_2_ requests, as an initial step, the development of adequate catalysts that can profit from the energy combination and can drive selectivity to a single product. In the catalysts field, a particularly effective formulation comes out from the use of composite catalysts. Compared with single phase catalysts, composite catalysts could present a series of advantages. Classically, they were utilized to increase surface exposure of active species but, also, can provide specific novel catalytic (redox, acid–base, etc.) properties evolving from the (sub)nanometric nature of specific phases as well as from interfacial effects, both leading to the appearance of new active sites.^26^ In addition, for photo-based processes, the contact between phases can lead to efficient charge carrier separation after light excitation, facilitating the subsequent use of charge carrier species in chemical steps.^27,28^

In this work, we explored the activity of subnanometric and nanometric InO_*x*_ species in intimate contact with nanoplatelets of TiO_2_. Both oxides present activity in the CO_2_ valorization under dual-excitation. The presence of nanostructured species and defects on both semiconductors were highlighted as critical properties to control the surface and catalytic properties, particularly in the case of indium-based systems.^29−32^ Nonetheless, the combination of these two active oxides has, to our knowledge, not been explored to date in this dual process and would provide different active species in comparison to those previously tested in the single-oxide materials. In fact, we will see that the nature of the chemical species produced by a strong interfacial interaction changes dramatically as a function of the indium content and that this leads to a strong modulation of the catalytic activity. To measure quantitatively the functional properties of the solids under single and dual excitation, we carefully explored the synergy of the energy combination. As will be shown, the InO_*x*_–TiO_2_ composite system is at the top of the known thermo-photo systems, proving at the same time a highly selective system, with the production of CO as a single carbon-containing product. The reverse water gas shift (RWGS) can be considered as a first step in the conversion of carbon dioxide where CO production occurs. The CO molecule is a valuable compound for the synthesis of a large number of different chemicals.^33^ Particularly, the RWGS is a key step in obtaining C1 and C2+ products.^34,35^ As the main result, this work uncovers new, highly active, selective, and stable indium-based species and provides a structure–activity relationship rationalizing the functional properties.

## 2 Experimental Section

### 2.1 Catalyst Preparation and Characterization

The preparation of the catalysts was carried out using the reverse microemulsion method. First, two organic phases were prepared with the same composition: *n*-heptane, Triton X-100 as surfactant, and hexanol as the cosurfactant. The microemulsion utilizes a water-to-surfactant ratio of 18. After sonication for 30 min, a solution of indium(III) nitrate hydrate (0.5 M) and water was added to the first one, while another solution of tetramethylammonium hydroxide (TMH; 1 to 1 ratio with indium) and water was added to the second one. Both solutions were further sonicated for 1 h. After this, the one containing TMH was overturned on top of the other in order to precipitate the indium containing species. After sonicating the new solution for 3 min, the titania precursor was added to it drop by drop. The mixture was left stirring overnight. After 24 h, the mixture was centrifuged, washed, and dried for 12 h in an oven at 100 °C. Once ground, it was calcined for 2 h at 500 °C.

Catalysts were subjected to extensive characterization. Chemical composition (cation content) was analyzed with the help of inductive couple-plasma optical emission spectroscopy (ICP-OES; Optima 3300DV PerkinElmer spectrometer, USA) and X-ray diffraction (XRD). The use of a Bruker D8 Advance diffractometer with Ni-filtered Cu Kα radiation (λ = 0.15406 Å) rendered the XRD patterns of the solids under examination. Raman spectroscopy was utilized to interrogate the samples with the help of Jobin-Ybon HR320 apparatus and the 614.6 nm He excitation line. UV–visible spectra were obtained using a Varian Cary300 apparatus (USA) and utilized to measure the band gap energy. Catalysts were also subjected to degassing overnight at 140 °C to test the morphological properties. To this end, nitrogen physisorption experiments were carried out with Micromeritics Tristar-II 3020 equipment, USA. PL experiments utilized an excitation line of 365 nm and a Horiba FluoroMax Plus (Germany) fluorescence spectrophotometer. Photoelectrodes were prepared by coating FTO pieces (ca. 1 × 2.5 cm) with the studied photocatalysts. The catalyst loading and coating procedure were the same described in the Supporting Information section to carry out catalytic tests. The coated area for the In_2_O_3_, TiO_2_, and 2.5 InTi samples takes values of 1.73, 1.71, and 1.74 cm^2^, respectively. The photoelectrochemical characterization was performed by using a Gamry Reference 3000 Potentiostat/Galvanostat/ZRA and 500 W Xe lamp as a radiation source (Quantum Design). The measurements were performed in a three-electrode setup, using the photoelectrodes as working electrodes, Ag/AgCl (3 M) as the reference electrode, and a Pt wire as the counter electrode. A Na_2_SO_4_ (Aldrich, ACS reagent, > 99%) 0.1 M solution was used as electrolyte, whose pH was 7.3. The photoelectrode was irradiated back-face and it was placed facing the counter electrode. Linear sweep voltammetry (LSV) experiments were carried out from +1 V Ag/AgCl to −0.6 V vs Ag/AgCl with a scan rate of 5 mV s^–1^ and chopping irradiation with an on/off frequency of 20 s. Nyquist plots were obtained by performing electrochemical impedance spectroscopy (EIS) using a sinusoidal perturbation between 100000 and 0.2 Hz with an AC amplitude of 10 mV. The applied potential was +1 V vs Ag/AgCl.

Transmission electron microscopy (TEM) was carried out using a 200 kV JEOL STEM 2100. The microscope was equipped with an energy-dispersive X-ray spectrometer (EDS) to evaluate the composition. For the TEM histograms, approximately 60 images of each sample were evaluated to study the particle size and morphology were representative. XPS experiments were carried out using a SPECS spectrometer (UK). The spectrometer is equipped with a PHOIBOS 150 WAL hemispherical energy analyzer, an XR 50 Al-X-ray source, and a μ-FOCUS 500 X-ray monochromator. To take into account the effect of potential charging effects, the binding energies (BE) were referenced with the help of the C 1s peak (284.6 eV). The CASA 2.3.25 software was utilized for fitting experimental data. A Shirley background allowed the elimination of inelastic and other undesired contributions.^36^

X-ray absorption spectroscopy (XAS) measurements were conducted at the 8-ID beamline at the NSLS-II of Brookhaven National Laboratory. The X-ray absorption near-edge structure (XANES) spectra and the extended X-ray absorption fine structure (EXAFS) spectra at the In K-edge (27940 eV) were acquired through fluorescence mode, using a total fluorescence yield detector (PIPS diode). The beamline’s X-ray optics comprise a collimating mirror system with a Pt/Si/Rh coating, a high heat load Si(111) monochromator, a focusing toroid mirror with Pt/Rh coating, and the high harmonic rejection mirror system (Rh/Si/Pt) situated in the endstation. Further focusing is achieved through a policapillary lens in the endstation. The powder samples were dispersed on Kapton tape during the measurement, and the energy calibration was conducted using In foil simultaneously measured with the sample. The phase and amplitude functions for In–O and In–In were derived from home-prepared In_2_O_3_ material (purity checked by XRD), exhibiting a cubic structure of Bixbite-type with space group *Ia*3̅. The data processing was carried out utilizing the IFEFFIT package.^37^

Samples were named *x*InTi, where *x* corresponds to the content of indium expressed as the molar percentage of the total cation content. As measured using ICP-OES, the indium content of the solid(s) corresponds to the nominal content within an error below 3.4%.

### 2.2 Activity Measurements and Quantitative Analysis of Activity

Catalytic activity and selectivity parameters were measured using a thin film prepared by homogeneous distribution of the catalyst(s) in a Pyrex glass cylinder. The thin film ensures that the interaction with temperature and light can be carefully controlled, measured, and mathematically modeled. A coaxial, double jacketed configuration was used as the reactor. Inside the inner cylinder, a compensated resistance was used to heat and control temperature. The catalyst is placed on the outer side of the inner cylinder. Outside the outer cylinder, the illumination source consists of four lamps symmetrically positioned on a rectangular box. Gases can pass between the two Pyrex cylinders and contact the catalyst under controlled temperature and illumination conditions. Figure S1 in the Supporting Information section provides details about the reactor configuration. The reaction mixture of CO_2_, H_2_, and N_2_ (10 mL min^–1^) was built up with a CO_2_:H_2_ ratio of 1:1. ^13^C labeled carbon dioxide, supplied by Sigma-Aldrich (99% purity), was utilized in selected experiments. Experimental conditions to test the catalysts scanned the temperature between room temperature (RT) and 350 °C, without and with simultaneous illumination. Gas chromatography (Agilent GC 7890) and mass spectrometry (OmniStar 300, Pfieffer) allowed the analysis of the reaction output. Full details of the experimental step-up, including light and heating source characteristics, gas handling and environment, catalyst deposition, reactants/product detection systems, and experimental procedures, are detailed in the Supporting Information section.

The reaction rate (*r*) was the initial parameter analyzed to compare the catalysts. This was carried out following the rules established by the IUPAC.^38^ The specific procedure is described in the Supporting Information section. Rates under thermal-alone (hereafter described by the *T* suffix), photoalone at room temperature (*P* suffix), and thermo-photo (*TP* suffix) were obtained. Following previous research, to provide an estimation of the synergy between the two excitation sources, kinetic, global energy, and photorelated parameters were calculated.^39^ First, an excess rate (*r*_*e*_) was calculated as quoted in eq 1.
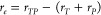
1

The excess rate was expressed herewith as a percentage over the *r*_*TP*_ parameter. The quantum efficiency of the reaction was also investigated and calculated to provide insights into the usage of photons by the catalysts. Such a calculation requires measuring and obtaining the local superficial rate of photon absorption. In the dual excitation, it takes into account thermal effects on optical properties as detailed in the Supporting Information section.^39^

A third parameter follows the commonly utilized global energetic efficiency of the process.^22^ This parameter is measured with the help of eq 2.
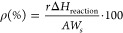
2Here, *r* is the rate of the reaction (mol s^–1^) and Δ*H*_reaction_ is the standard enthalpy of the reaction. Note that the formation of CO takes place through the reverse water gas shift reaction. This reaction is an equilibrium, and this point should be considered in the calculation to obtain values for the denominator in eq 2. Full details of the numerical procedure are presented in the Supporting Information section. Finally, the *W*_*s*_ parameter corresponds to the total energy supplied by the dual excitation per surface area unit (W m^–2^) and *A* is the surface area of the catalyst.^22^

## 3 Results and Discussion

**Figure 1:**
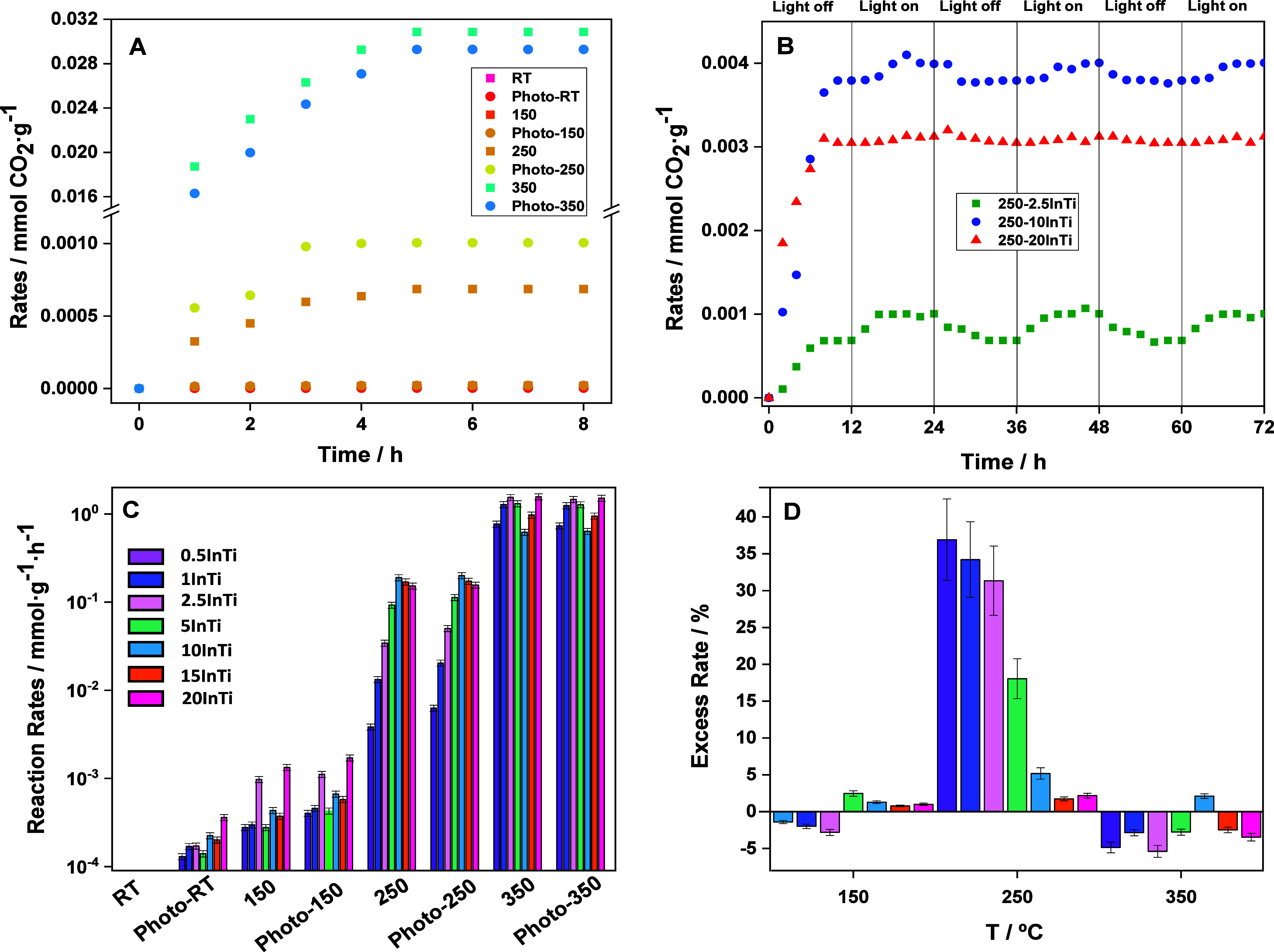
(A) Time of course of the reaction for the 2.5InTi sample. Photo, thermal, and thermo-photo conditions at different temperatures are presented. (B) Long time of stream test under light on–off conditions for 2.5InTi, 10InTi, and 20InTi samples. Thermo-photo conditions at 250 °C. (C) Reaction rates for InTi samples under photo, thermal, and thermo-photo conditions. Note the logarithmic scale of the OY axis. (D) Excess rates for InTi samples under thermo-photo conditions.

The activities of the materials under single and dual excitation conditions are described in the plots of Figure 1. CO is the single product of the reaction, which thus takes place through a reverse water gas shift mechanism. Catalysts showed a selectivity exceeding 98% for all cases. Figure S3 displays a kinetic experiment using isotopically marked CO_2_ to demonstrate that this reactant is the source of the already mentioned reaction product under pseudostationary conditions. Figure 1A contains examples of the catalytic output using the 2.5InTi sample. After an initial (slow) period of transient response, the activity reached stationary conditions. The activity is rather low (although not negligible) under photo excitation at room temperature, thermal, and dual thermo-photo conditions up to 150 °C. A significant rise in activity is observed under single (thermal) and dual (thermo-photo) conditions at 250 °C. The higher activity under dual excitation with respect to thermal-alone excitation can be noticed. A further, significant boost of activity is also detected for both conditions at 350 °C. Note, however, that such a boost decreases the difference between the thermal and thermo-photo response of the solid, presenting rather limited differences. The activity of the solids has been further investigated under long-term operation conditions. Figure 1B displays representative examples of thermo (called light-off on the figure) and thermo-photo (light-on) conditions at 250 °C. The plot allows the analysis of the response of three solids, 2.5InTi, 10InTi, and 20InTi, for continuous operation up to 72 h. The lower response under thermal-alone conditions as well as a reversible response can be observed for all solids. To facilitate the analysis of the catalytic response, in Figure 1C,D, we present the normal and excess reaction rates for the samples, respectively. The activity of the two single cation oxides (TiO_2_, In_2_O_3_) used here as reference systems is about 1 order of magnitude inferior to the lowest In containing composite sample presented in Figure 1C, as reported previously for the case of titania.^32^The reaction rate in these two panels of Figure 1 quantifies the strong temperature dependence of the catalytic response presented in Figure 1A,B for representative examples. Note that rates included in Figure 1C spread over ca. 4 orders of magnitude. As mentioned for the 2.5InTi sample, the catalytic output is relatively low for single (photo and thermal) and dual excitation conditions below 150 °C. After this temperature, the reaction rate grows quickly with the In content of the materials up to the 5InTi sample, presenting some leveling off for samples having higher indium loadings. At 350 °C, the activity shows a less marked dependence on the In content.

The comparison between the catalytic response under thermal and thermo-photo conditions is better visualized using the excess reaction rates presented in Figure 1D. Rather limited variations are observed until 150 °C. At 250 °C, the beneficial effect of using dual thermo-photo excitation is clearly demonstrated. An excess of 33–37% is observed for samples with In loading below or equal to 2.5InTi. The excess rate decays significantly after this sample, with 5InTi displaying a transition response. At 350 °C, with the boost of activity, the beneficial thermo-photo effect has disappeared. Thus, as outlined, the activity of the samples in the CO_2_ valorization shows a strong dependence on the In content. Activity increases with the In content, but the beneficial effects of using dual thermo-photo conditions appear to decrease significantly for loadings above that of the 2.5InTi sample. To put into context the performance of the materials, we note that the 2.5InTi and 10InTi samples, representative of the low and high loading regions, show reaction rates of 0.05/1.54 and 0.59/1.67 mmol CO_2_ g^–1^ h^–1^, respectively, for 250/350 °C. To our knowledge, the excess rate of ca. 33%, corresponding to the first sample, is the maximum presented in the literature for the carbon dioxide thermo-photo valorization reaction.^22,32^ Moreover, the global efficiency parameter (eq 2) takes maximum values of 0.33 and 1.31 for the 2.5InTi and 10InTi systems. These values allow a quantitative comparison with previous literature reports and can be compared with the benchmark value of 0.83% reported previously for a Ga–Cu/CeO_2_ catalysts under dual excitation at 250 °C.^22^ For pure In_2_O_3_, nanostructured (nanosheets) and/or black materials have been previously tested in the thermo-photo valorization of CO_2_.^29,30,40^ The materials presented high selectivity to CO, although with higher traces of CH_4_ than that here presented. Unfortunately, only reaction rates were reported, without calculation of the global efficiency. The comparison with our catalysts would thus lack a quantitative basis due to significant differences in experimental conditions concerning light conditions as well as material′s physicochemical properties. For nanosized TiO_2_, the calculation of the efficiency parameter was previously reported under similar experimental conditions and rendered a value below 0.1%.^31,32^ A summary of literature references reporting activity with the help of the above-mentioned (activity) parameters is presented in Table S1 of the Supporting Information section. From this comparative analysis, it could be noted that the advantages of using a composite InOx/TiO_2_ system with respect to previously tested (single oxide, back) or benchmark materials become evident in terms of efficiency reached under the binary thermo-photo excitation. Also, compared with our nanosized single oxide reference systems, the combination of oxide-type components boosts efficiency significantly, above 1 order of magnitude.

**Figure 2:**
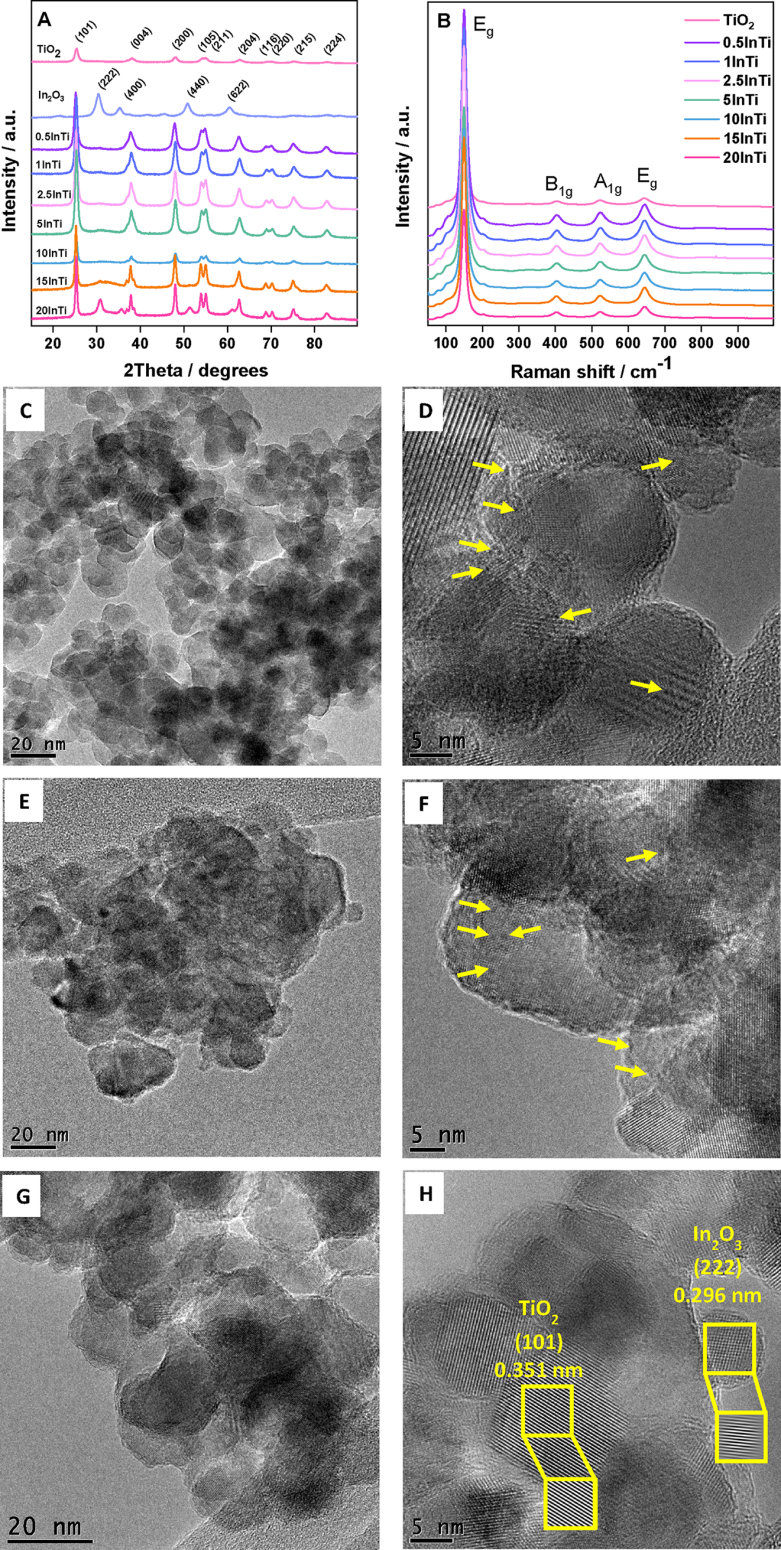
(A) XRD patterns for the InTi and reference samples. (B) Raman spectra for the InTi and reference samples. Labels for anatase peaks are included. (C,D) TEM Micrographs for the 2.5InTi sample. (E,F) TEM Micrographs for the 10InTi sample. (G,H) TEM Micrographs for the 20InTi sample. Arrows mark indium-containing particles.

A complete characterization analysis was carried out to interpret the physicochemical grounds for the catalytic behavior. In particular, a critical point to analyze concerns the behavior of the series as a function of the In content of the sample and the substantial differences between the low and high loaded samples outlined above. As a first step in this quest, the structural properties of the solids were analyzed with the help of several techniques. Results are summarized in Figure 2. XRD patterns of the samples are presented in panel A of the figure. The reference single cation oxides are also included, and corresponding patterns are indexed. The comparison with the reference pure oxides indicates the dominance of the anatase pattern (space group I41/amc; JCPDS card 21–1272) in the XRD signal of all samples. The peaks associated with indium oxide (space group *Ia*3̅; JCPDS card 06–0416) appeared only for the 15InTi and 20InTi samples. The dominant presence of anatase is also detected in the Raman spectra, with the absence of any signal associated with the indium component.^41^ The anatase component presents a primary particle size, as collected in Table 1 for all samples. The InTi catalysts display a higher primary particle size and lower BET surface area than the two single oxide reference systems. Within the series of samples, a somewhat constant BET surface area, from ca. 92 to 82 m^2^ g^–1^, and higher primary particle size of anatase, going from 10 to 19 nm, can be observed with the growth of the indium content of the composite solids. The different anatase primary particle sizes and the surface presence of indium entities are reflected in the textural properties of the materials, as evidenced by the nitrogen adsorption–desorption isotherms (Figure S4). Catalysts (and reference materials) display hysteresis loops characteristic of mesoporous materials. According to the IUPAC, the pure TiO_2_ sample displays an H2(a) type hysteresis loop.^42^ The InTi catalysts appear to be dominated by the anatase phase morphological (textural) properties, in accordance with its dominant contribution by weight. However, the similitudes with pure indium oxide appear to be more evident in the hysteresis loop as the In content of the sample increases. Overall, the textural parameters indicate that indium contact with the dominant phase (anatase) increases the primary particle size of the latter and alters the pores’ mouths of anatase. This, however, does not significantly affect the surface area of the solids as a function of the In content, a fact which may be directly connected with changes in the connectivity of the anatase nanoplatelets, which may counteract the surface effects derived from the contact between the indium and titanium containing components and the potential diminishing of surface the area directly linked with the primary particle size increase of anatase (the dominant component).^43^

**Table 1:** Main Physicochemical Parameters for the Samples^a^

**sample**	**BET area** (m^2^/g)	**pore volume** (cm^3^/g)	**particle size (nm)**	**band gap (eV)**
TiO_2_	146	0.17	8.9	3.06
0.5InTi	92	0.15	10.3	3.10
1InTi	91	0.14	11.3	3.07
2.5InTi	89	0.16	12.1	3.03
5InTi	91	0.15	11.8	3.03
10InTi	95	0.15	14.3	3.04
15InTi	97	0.16	16.6	3.03
20InTi	82	0.18	18.9	3.06
In_2_O_3_	146	0.46	6.2	2.66

a Results for pre/(thermo-photo) post-reaction catalysts are included. Primary particle size reported corresponds to the anatase component in the case of the InTi catalysts. Average standard error: BET area 7.6%; particle size 1 nm; band gap energy 0.03 eV.

**Figure 3:**
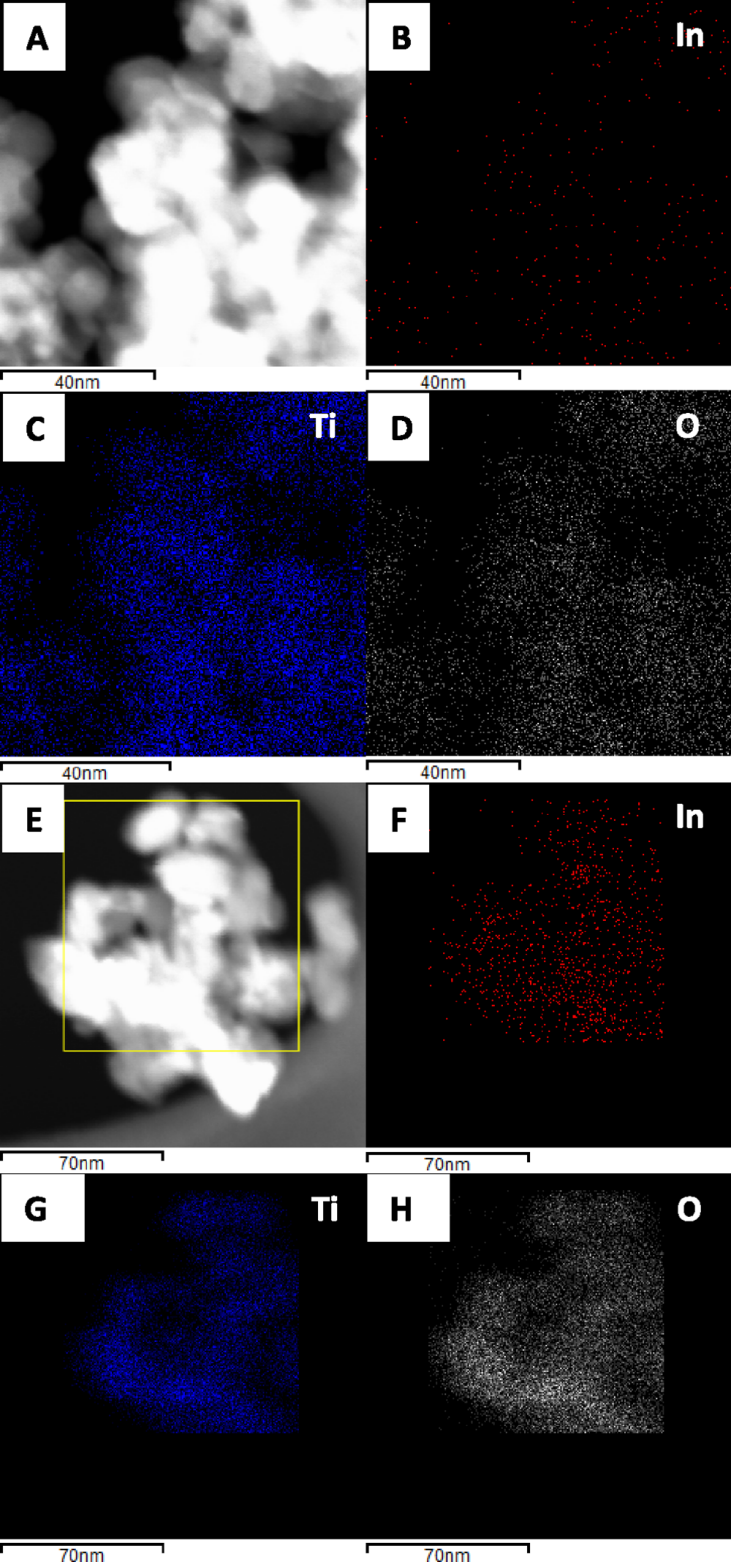
Dark field STEM images and XEDS mapping for the 2.5InTi (A–C) and 10InTi (E–H) samples.

To obtain structural information for the indium containing component, a thorough microscopy study was carried out. Figure 2 contains low- and high-magnification micrographs for representative samples. In all cases, the anatase is observed forming nanosized platelets, which grow in primary particle size with the indium content of the material, in agreement with the XRD results. For the low loading 2.5InTi sample (Figure 2C,D), indium containing entities with a size of less than 1 nm can be detected. The XEDS analysis provides proof of the presence of In at the surface of anatase as well as the good dispersion achieved using our microemulsion method (Figure 3). For the 10InTi samples, indium containing particles with an average particle size of ca. 1 nm were observed. The corresponding primary particle size distribution is shown in Figure S5. For indium loadings below or equal to 10 mol %, the particle size distribution provides evidence of the rather limited size of the indium containing entities as well as the significant homogeneity achieved on the anatase surface. This is further evidenced by the XEDS analysis presented for 2.5InTi and 10InTi samples in Figure 3. Indium loading higher than that of the 10InTi leads to a significant increase of size. Figure 2 contains micrographs of the 20InTi sample. Well-defined In_2_O_3_ oxide-type entities can be detected with the help of electron diffraction (panel G). This obviously occurs with a significant increase in the primary particle size, as quantitatively measured using the data presented in Figure S5 for the particle size distribution of the 20InTi catalyst. From the microscopy study, we can confirm that an extreme control of the particle size is reached for samples with loadings below 2.5InTi. Subnanometer entities appear characteristic of such low loading samples. Increasing the indium loading up to 10InTi generates nanometer-size indium particles with a rather modest increase of the average primary particle size, consistently around ca. 1 nm. Above this sample, the growth of the mentioned parameter becomes evident.

**Figure 4:**
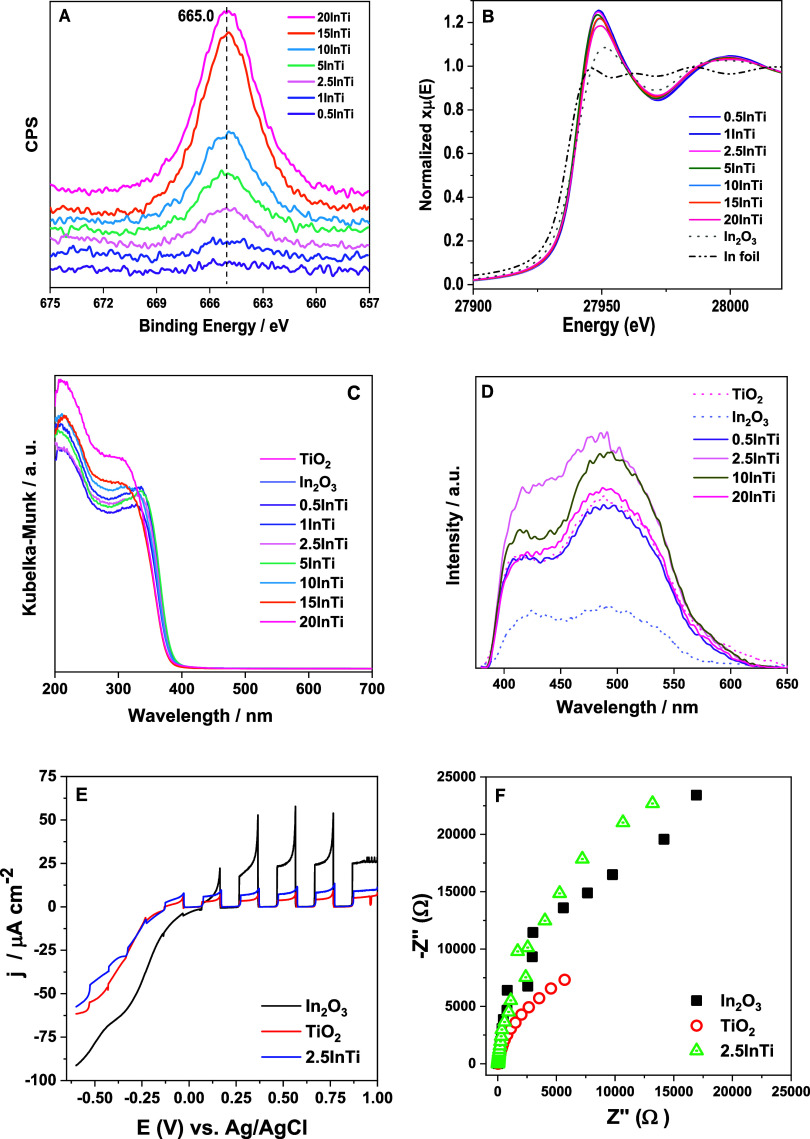
(A) In 3d_5/2_ XPS spectra for the InTi samples. (B) XANES spectra for the InTi catalysts and references samples. (C) UV–visible plot spectra for the InTi samples. (D) PL spectra for selected InTi samples. Excitation at 365 nm. (E) Linear sweep voltammograms under chopped irradiation recorded from +1 V vs Ag/AgCl to −0.6 V vs Ag/AgCl at a scan rate of 5 mV s^–1^. (F) Nyquist plots obtained from the EIS measurements performed at +1 V vs Ag/AgCl from 100000 to 0.2 Hz.

The electronic properties of the indium entities were also studied by using a multitechnique approach. Both XPS (Figure 4A) and XANES (Figure 4B) techniques show the presence of In^3+^ in our samples. The In 3p_3/2_ XPS signal shows the same binding energy of ca. 665.0 eV for all samples, while the XANES signal of the catalysts displays an invariant energy of the absorption edge at ca. 27938.0 eV.^44,45^ The presence of the In^3+^ oxidation state is an expected result. Nonetheless, some differences can be noticed in the XANES spectra of the materials. The intensity of the continuum resonances appearing in the XANES spectra and, particularly the white line, are strongly sensitive to the electronic state of the solids.^45^ The comparison with the In_2_O_3_ reference sample shows that although there is no change in the oxidation state with respect to the mentioned reference single oxide, the low-lying states near the continuum (the conduction band) have lower electronic density with respect to the bulk reference. This is a proof of a strong In–Ti interaction and/or a strong size effect that may not be detected using other techniques. This is further discussed at length below. Before doing it, we present the UV–visible spectra of the samples (Figure 4C). The samples show a relatively similar spectrum profile, with relatively modest variation between them. Considering that anatase, the main component of the solids, is an indirect gap semiconductor, we calculated the band gap energy of the samples.^46^Table 1 collects the corresponding data, displaying results with few differences between samples as well as with respect to the anatase reference.

In addition, the photoluminescence (PL) spectra of the samples were recorded and presented in Figure 4D. The PL spectroscopy provides information about the de-excitation of charge carriers after light excitation and the corresponding electronic levels involved.^47^ The comparison with the reference single oxide systems indicates that the PL signal of the InTi samples is dominated by the anatase de-excitation channels. Two typical peaks are observed. They are called green and red and are located at ca. 425 and 500–550 nm. Their presence indicates the existence of localized states near both the conduction and the valence band and associated the defect-type centers.^46^ Samples do not display significant differences between them or with the anatase reference system in terms of the shape profile, although variation of intensity can be observed, with a maximum for the 2.5InTi sample. An increase in the PL intensity can be correlated with a higher charge carrier recombination, and thus a lower probability of being involved in chemical reactions for charge carrier species formed after light excitation.^46,48^ On the other hand, no correlation can be observed between the optimum thermo-photo effect (Figure 1) and the charge carrier recombination capability of the samples (Figure 4D).

Linear sweep voltammetry tests were performed under chopped irradiation to further evaluate the photocurrent response, as it gives information about the photogeneration and separation of charge carriers. As the photoelectrode was irradiated back-face, most of the charge carriers are photogenerated close to the catalyst-FTO interface, so electrons will be swiftly transferred to the FTO while holes will migrate toward the semiconductor-electrolyte interface.^49,50^ The steady-state photocurrent detected for the In_2_O_3_, TiO_2_ and 2.5InTi were ca. 25.0, 5.5, and 9.0 μA cm^–2^ respectively, see Figure 4E. The initial spike was ascribed to the charge of the space charge capacitance due to the separation of the charge carriers.^51^ The photocurrent produced by the In_2_O_3_ reference was over 4.5 and 2.7 times greater than the values obtained with TiO_2_ and 2.5InTi, respectively. For all the catalysts, the photocurrent response was reproducible over the on/off cycles, becoming quickly zero when the light is switched off and reaching the maximum photocurrent as soon as the light is on. Therefore, these results seem to indicate that the electron–hole separation is more efficient in the In_2_O_3_ catalyst followed by 2.5InTi and TiO_2_. Note that the results further support the absence of correlation between charge carrier separation and catalytic activity, as previously discussed using photoluminescence measurements. On the other hand, Nyquist plots (Figure 4F) were obtained from the EIS experiments to evaluate the electron-transfer process at the semiconductor–electrolyte interface.^52^ Since a smaller semicircle radius indicates a smaller charge transfer resistance, it can be concluded that the TiO_2_ reference exhibits the fastest charge transfer followed by In_2_O_3_ and TiO_2_.

**Figure 5:**
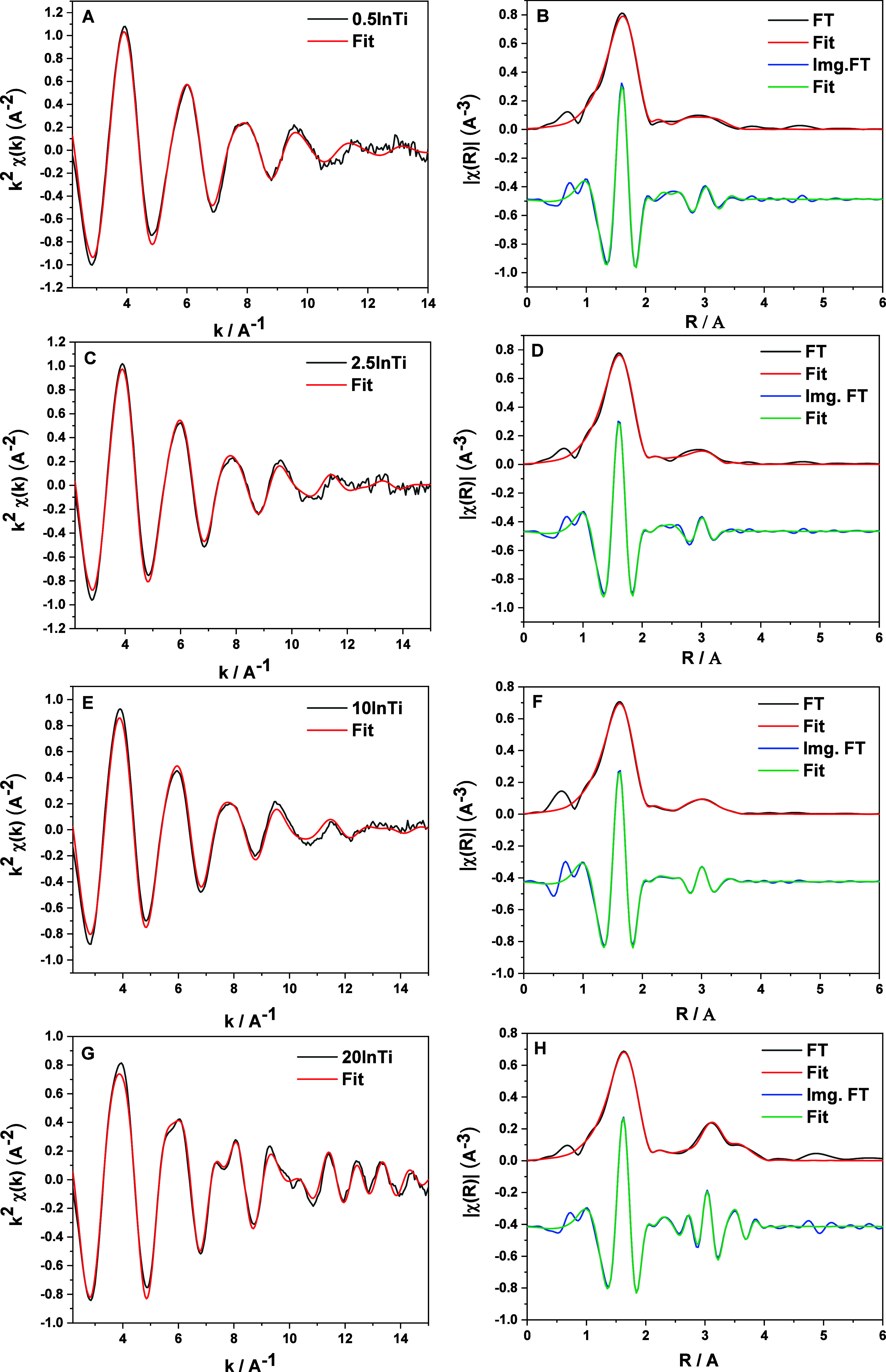
*K*^2^-weighted EXAFS signals (A,C,E,F) and imaginary part and module of the Fourier transform (B,D,F,H) for selected samples. Experimental signals and fitting results are presented. (A,B) 0.5InTi, (C,D) 2.5InTi, (E,F) 10InTi, and (G,H) 20InTi.

The study of the electronic properties thus shows little alternation or variation along with the indium loading of the samples. Moreover, light-related effects connected with charge recombination and handling do not appear to control the promotion of activity under dual thermo-photo conditions. Therefore, it is possible that structural differences between samples would drive activity. A detailed EXAFS study was then conducted to shed light on the question and provide information about the local order around indium entities. Rather good (high single-to-noise ratio) EXAFS signals were recorded and presented in Figure 5. The high *k* and *R* ranges available for fitting allow us to fit up to 5 shells. According to the Nyquist theorem and using the ranges presented in Table 2, we obtain a (minimum) number of free parameters of 21.^53,54^ Fitting was carried out using *k* and *R* signals weighted in *k*^1^/*k*^2^/*k*^3^. For the low loading samples, up to the 2.5InTi sample, Table 2 shows the presence of three different shells. A Ti–O shell with a coordination number above of that of pure In_2_O_3_ (CN = 6).^55^ Moreover, the samples show the presence of an In–Ti shell at ca. 3.60 Å, indicating the strong interaction between the two components of the composite solids.^56^ In addition, a rather short In–In distance, absent in the single oxide system, can be detected (Table 2). The coordination number of the latter (ca. 0.5) indicates that our samples contain an “equimolecular” mixture of isolated In ions or In–In pairs in oxidized environments, located onto the anatase surface and forming strong heterocation bonds. The limited atomicity of these species justifies the short In–Out distance detected.

**Table 2:** EXAFS Fitting Parameters^a^

**shell**	***R*/Å**	***N***	**Δσ**^**2**^**/Å**^**2**^	***E***_**0**_**/eV**
0.5InTi k: 2.42–13.57 Å ^–1^; *R*: 0.92–3.57 Å				
In–O	2.11	7.1	8.4 × 10^–3^	2.8
In–In	2.92	0.5	1.1 × 10^–2^	2.8
In–Ti	3.61	2.0	1.2 × 10^–2^	2.8
1InTi k: 2.46–13.58 Å ^–1^; *R*: 0.89–3.43 Å				
In–O	2.12	7.1	8.4 × 10^–3^	2.6
In–In	2.89	0.4	8.5 × 10^–3^	2.6
In–Ti	3.59	2.2	1.2 × 10^–2^	2.6
2.5InTi k: 2.44–13.58 Å ^–1^; *R*: 0.91–3.53 Å				
In–O	2.13	6.9	8.5 × 10^–3^	2.0
In–In	2.95	0.4	7.8 × 10^–3^	2.0
In–Ti	3.58	2.3	1.3 × 10^–2^	2.0
5InTi k:2.42–13.53 Å ^–1^; *R:* 0.92–3.54 Å				
In–O	2.12	6.0	8.1 × 10^–3^	1.9
In–In	2.93	0.4	9.5 × 10^–3^	1.9
In–In	3.31	0.5	1.6 × 10^–2^	1.9
In–Ti	3.59	1.3	1.0 × 10^–2^	1.9
10InTi k: 2.42–13.96 Å ^–1^; *R*: 0.92–3.59 Å				
In–O	2.12	6.0	8.1 × 10^–3^	2.1
In–In	2.86	0.4	1.9 × 10^–2^	2.1
In–In	3.30	0.7	6.9 × 10^–3^	2.1
In–Ti	3.62	1.3	1.1 × 10^–2^	2.1
15InTi k: 2.43–13.97 Å ^–1^; *R*: 0.93–3.63 Å				
In–O	2.12	6.2	8.3 × 10^–3^	2.3
In–In	2.92	0.3	8.9 × 10^–3^	2.3
In–In	3.31	0.9	1.6 × 10^–2^	2.3
In–Ti	3.60	0.8	9.5 × 10^–3^	2.3
20InTi k: 2.43–13.97 Å ^–1^; *R*: 0.93–3.63 Å				
In–O	2.12	6.0	8.2 × 10^–3^	2.7
In–In	3.36	3.0	7.0 × 10^–3^	2.7
In–In	3.85	0.9	3.9 × 10^–3^	2.7

a Sample name together with *k* and *R* fitting ranges are presented. Average standard error: distance 0.01 Å: CN 9.9%; Debye–Waller 11.2%; *E*_o_ 0.4 eV.

Subtle changes in the signals are observed from 2.5 to 10InTi sample. We attempted to fit the (5InTi, 10InTi, and 15InTi) samples using the 3 (In–O, In–In, In–Ti) shells utilized for the low loading samples but cannot provide reliable results. An additional shell was required, as summarized in Table 2. To analyze the statistical significance of using models with different numbers of shells, we carried out an *F*-test of the variance (summarized in eq S16 of the Supporting Information section) corresponding to the 3- and 4-shell fitting results for the samples mentioned. A representative example of the statistical test is presented in Figure S6.^54^ These *F*-tests show that the 4-shell model presented in Figure 5E,F improves the variance of the fitting with a probability above 97. Three % for the 5InTi, 10InTi, and 15InTi catalysts. The adequacy of the 4-shell model is thus accepted using the typical 95% cutoff decision level. For these three samples, the appearance of a new In–In shell at ca. 3.30–3.31 Å is indicative of the formation of clusters with a first cation–cation distance characteristic of a single oxide-type local environment.^55^ Nevertheless, the coordination number of such a shell is only ca. 8 (5InTi) to 15 (15InTi) % of the bulk. This fact and the absence of the ca. 3.85 Å In–In shell are indicative of the rather limited size of the new indium containing species present from the 5InTi to 15InTi samples (Table 2). This agrees with the size of ca. 1 nm observed using TEM for the 10InTi catalyst (Figure S5). On the other hand, the decrease of the In–Ti coordination number in the 15InTi sample makes this sample a limiting case with the highest loading tested (20 mol %).

Finally, for the 20InTi sample, we detected the presence of In–O (ca. 2.12 Å) and In–In (ca. 3.30–3.35 and 3.85 Å) shells characteristic of the indium oxide local environment.^55^ Note the concomitant lack of In–Ti contacts. Although the latter may exist, because EXAFS is a bulk-averaged technique, its presence cannot be detected if its molar contribution (to the In as X-ray absorbing center) is below ca. 10%. The outcome of the EXAFS analysis is consistent again with the microscopy results (Figure 2; Figure S4) that showed indium oxide clusters of about 5 nm for the 20InTi case.

**Figure 6:**
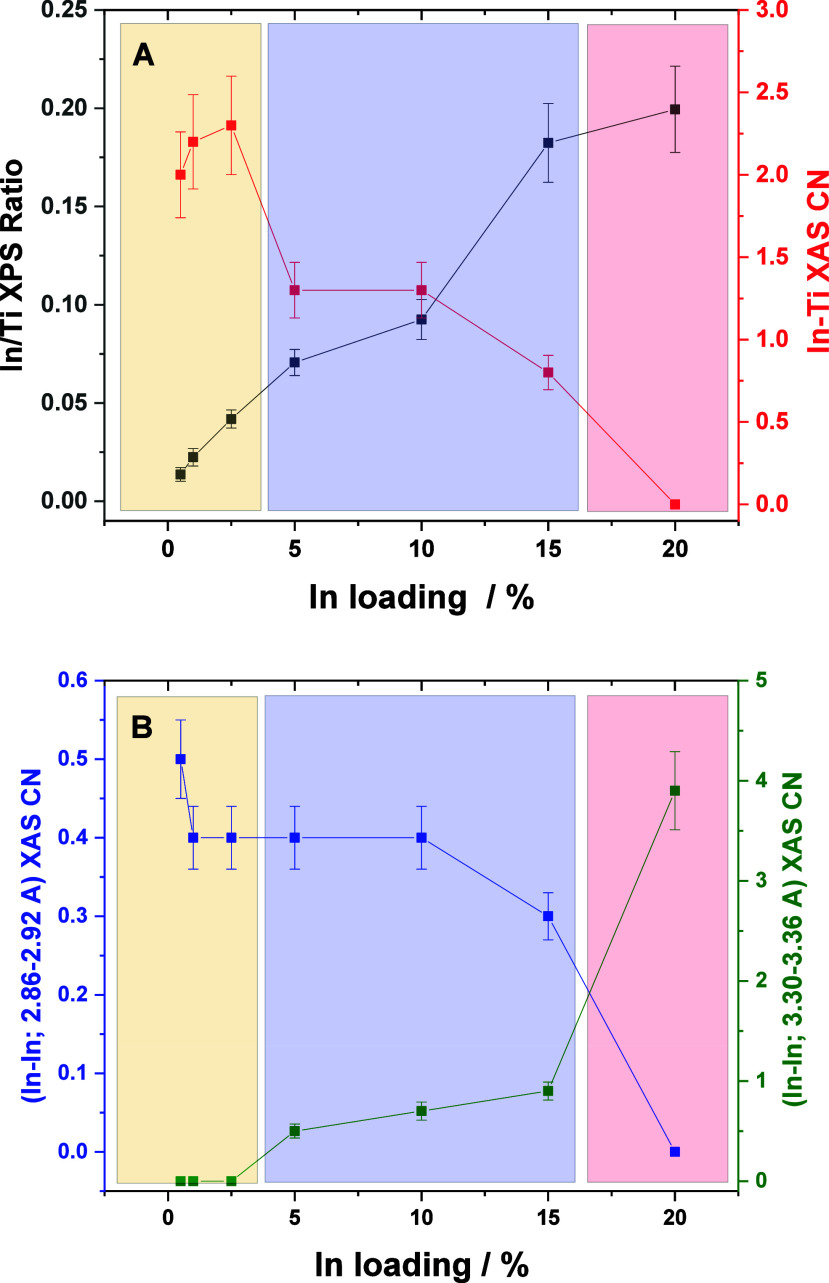
(A) In/Ti XPS atomic ratio and coordination number (CN) of the XAS In–Ti contribution as a function of the In loading of the samples. (B) Coordination number (CN) of two XAS In–In contributions as a function of the In loading of the samples. Lines connecting points are a visual guide.

The results discussed above clearly show that structural details can be at the core of the different catalytic behaviors of the samples. To investigate this point further, the coordination number of the In–Ti shell detected in the EXAFS analysis and the In/Ti XPS ratio are compared in panel A of Figure 6 for the different InTi materials. In accordance with the three types of indium containing species detected with EXAFS (and visualized in the figure with different colored boxes), we can see that both signals are consistent between them and would point out the “single” presence of (the EXAFS detected) oxidized, isolated In and In–In paired species for samples up to 2.5InTi. The extreme control of the In dispersion can be noticed. After this point, the XPS signal grows significantly up to the 15InTi. This is observed together with an important decay of the In–Ti coordination number (Figure 6A) as well as the appearance of the In–In shell at ca. 3.30–3.36 Å (Figure 6B). The low coordination number of the latter as well as the absence of the In–In shell at ca. 3.85 Å indicates that indium oxide type clusters with rather limited dimensionality are formed in 5InTi, 10InTi, and 15InTi samples.^57^ Thus, for these catalysts, two types of species considering (oxidized) isolated or paired indium containing species and oxide-type cluster entities with an average particle size of ca. 1 nm (according to TEM) coexist. The 15InTi sample appears as a limiting case for the situation encountered with the 20InTi sample, where the size of the indium oxide type cluster grows up to ca. 5 nm (TEM results) and the EXAFS results cannot detect any In–Ti interaction due to the limited presence of subnanometric or ca. 1 nm entities (Figure 6). Instead, the two In–In shells at ca. 3.35 and 3.85 Å, characteristic of well-defined indium oxide entities, are detected.^55^

**Figure 7:**
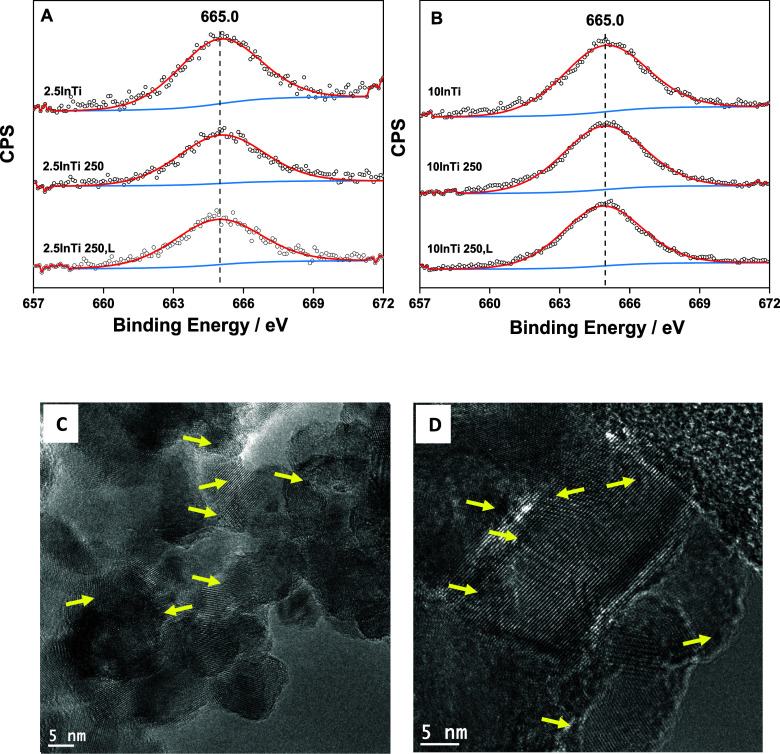
Results for 2.5InTi (A,C) and 10InTi (B,D) postreaction samples under thermo (XPS) and thermo-photo (XPS, TEM) conditions at 250 °C. (A,B) In 3p_3/2_ XPS results. (C) TEM micrograph for the 2.5InTi sample. (D) TEM micrograph for the 10InTi sample.

To end the discussion, it is important to emphasize that such indium containing species are stable under reaction conditions. In Figure 7, we collected XPS and TEM results for the 2.5InTi and 10InTi samples. These two samples exhibit activity characterized for relatively low thermal but high thermo-photo activity for the first case and, contrarily, high thermal but low thermo-photo activity for the second case. The stability under thermal-alone and thermo-photo conditions at the temperature where maximum thermo-photo activity is reached was proven, in the first place, using XPS. The absence of changes in the binding energy and the intensity of the signal can be highlighted in both cases. Furthermore, the TEM study also shows the stability of the materials, with the presence of subnanometer size species in the 2.5InTi case. For the 10InTi case, the analysis of the particle size distribution is included in Figure S5. This figure provides evidence of the lack of significant changes in the primary particle size distribution for this sample as well as the 20InTi catalyst. We stress that, as previously discussed, all indium containing species present in InTi catalysts have an invariant In^3+^ oxidation state, with relatively modest changes in the electron density occupancy as detected with XPS and XANES (Figure 4).

**Figure 8:**
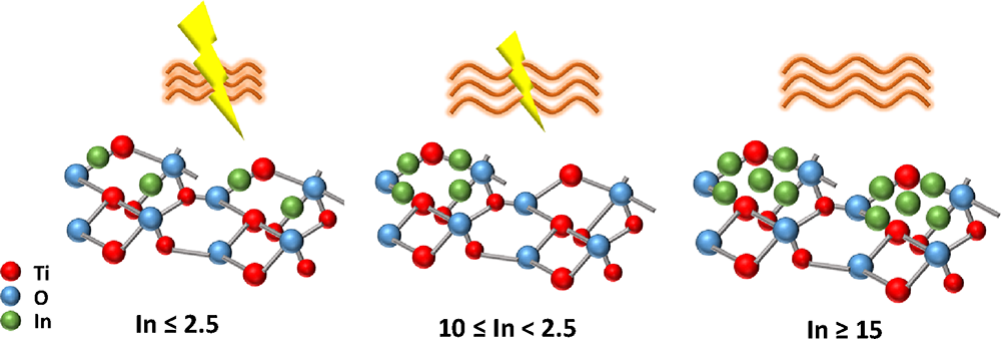
Schematic view of the indium containing surface species and catalytic properties as a function of the indium loading of the samples.

Thus, the size evolution and the concomitant loss of the interfacial In–Ti interaction drive the structural and electronic properties of the catalysts. To illustrate the evolution of indium containing surface species and the relationship with activity, Figure 8 displays models for the system, detailing the indium containing species present at the surface of the anatase platelets. We can conclude that as the size grows, drastic changes in the catalytic behavior are observed. For samples below 2.5InTi, the presence of (nearly atomically dispersed) isolated In and In–In pairs in an oxidized environment, with strong In–Ti interaction, leads to poor thermo activity but a high promotion of activity under dual thermo-photo conditions. For samples from 5InTi to 15InTi (being the last sample a transition case), we observed the additional presence of an indium oxide type cluster with dimensionality in the 1–2 nm range. The corresponding region from 5InTi to 15InTi is characterized by a substantial increase of thermo activity and a gradual decrease of the thermo-photo promotion of the catalytic activity. Finally, the 20InTi contains as dominant species (*Ia*3̅) indium oxide cluster-type entities of ca. 5 nm, with catalytic behavior characteristic of the bulk type samples. Highly dispersed oxidized indium of limited size (sub- and nanometric size up to around ca. 1 nm) would thus appear as a new way to the previously published black and/or nanosheet type materials^29,30,40^ to boost dual thermo-photo activity.

## 4 Conclusions

In this work, a microemulsion method is utilized to provide strong control of the spread of oxidized indium containing species onto anatase nanoplatelets. The resulting materials correspond to a high surface area and mesoporous solids. The complete physicochemical study showed the presence of subnanometric mono- and dimeric indium species for loadings below or equal to 2.5 mol %. Above this point, the presence of size-limited indium clusters of ca. 1 nm was also observed up to indium contents around 15 mol %. For high loadings, the dominant presence of a well-defined nanocluster of ca. 5 nm with a local environment closely related to the *Ia*3̅ structure of the bulk oxide can be observed. Electronically, all indium species display an In^3+^ oxidation state, with subtle differences between them in terms of the electron density as a result of the interaction with the anatase component and the concomitant high dispersion of the indium species.

The solids obtained show a strong dependence on their catalytic properties under dual (gas-phase, continuous) thermo-photo conditions for the valorization of carbon dioxide. Active, highly selective to CO, and stable response was observed for all catalysts. The functional properties showed a strong dependence on the structural properties of the indium containing species. Subnanometric species showed poor thermal activity, around 1 order of magnitude lower than the best samples. Yet an outstanding thermo-photo response was achieved, exceeding the sum of the thermo- and photo counterparts by above 30% and with the highest to-date reported energy efficiency of 1.31%. The thermal activity of the samples increases in the presence of 1–2 nm oxide-type clusters with strong interaction with anatase, but the thermo-photo effect decreases. The 5InTi sample appears as a compromise of a high thermo-alone activity together with reasonable thermo-photo efficiency (excess of ca. 17%). Samples having higher indium loadings display high thermal activity but near null thermo-photo promotion of the activity. We can thus observe the strong influence of the size and In–Ti interaction in the dual thermo-photo response of the solids. In short, subnanometric and nanometric (with no more than 1 nm) oxidized indium species supported on anatase provide highly active, selective, and stable solid(s) for the catalytic elimination and valorization of the carbon dioxide and can thus pave the way for new materials displaying advantages under dual excitation with respect to classical thermal processes.

## Supporting Information Available

Details about the catalytic setup, quantum efficiency calculation, optical measurements, and catalytic and characterization results (PDF)
