# Supplementary material for: Role of Atomicity and Interface on InOx-TiO2 Composites: Thermo-Photo Valorization of CO2
Source: ACS Appl Mater Interfaces. 2024 Jun 18;16(26):33461–74. doi: 10.1021/acsami.4c04803 (PMC11231977; doi:10.1021/acsami.4c04803)
Supplement: Supplementary file 1 — am4c04803_si_001.pdf [file am4c04803_si_001.pdf]

Supporting Information

Role of Atomicity and Interface on InO_x-TiO_2 Composites: Thermo-Photo Valorization of CO_2

*Rocío Sayago-Carro,[1] Irene Barba-Nieto,[2] Uriel Caudillo-Flores,[3] Álvaro Tolosana-Moranchel,[1] José A. Rodríguez,[2,4] Marcos Fernández-García,[*1] and Anna Kubacka[*,1]*

1) *Instituto de Catálisis y Petroleoquímica, CSIC.C/Marie Curie 2, 28049-Madrid, Spain.*
2) *Chemistry Division, Brookhaven National Laboratory, Upton, New York 11973 (USA).*
3) *Centro de Nanociencias y Nanotecnología, Universidad Nacional Autónoma de México, Ensenada, 22800, Mexico*
4) *Department of Chemistry, Stony Brook University, Stony Brook, New York 11794 (USA)*

Email: A.K. (ak@icp.csic.es), M. F-G (mfg@icp.csic.es)

Catalytic set-up and details

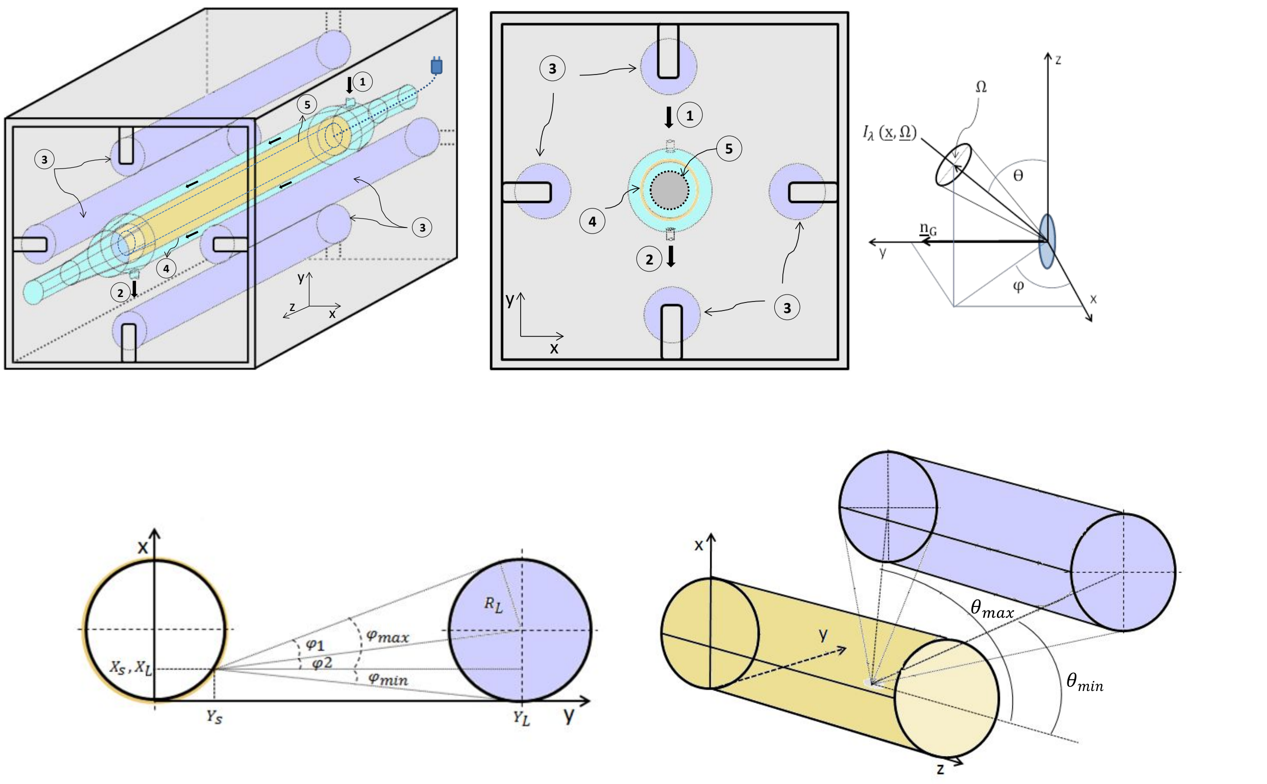

Figure S1. Upper, Left and Center: Photocatalytic annular reactor scheme; side and front views: (1) Gas inlet, (2) gas outlet, (3) UV lamp, (4) catalyst (brown) sample, (5) cartridge heater. q_{sup} Radiation flow on the surface of the sample (red), q_{n} radiation flow from the lamps (blue). Upper, Right: Center of coordinates located at the sample (defined by coordinates x_s, y_s, z_s). Down, Coordinate system to define the integration limits of the radiation Model. (Left) φ_{min} and φ_{max}. (Right) Θ_{min} and Θ_{max}.

The thermo-photo activity of the samples for methanol reforming was tested using a gas-phase continuous flow annular thermo-photo-reactor (pyrex) schematically depicted in Figure S1. The catalyst (ca. 0.2 mg cm^{-2}) was deposited onto the inner tube (ca. 15 cm; 0.8 cm diameter) as a thin layer from a suspension in ethanol. As previously detailed, a continuous rotation of the inner

glass tube during deposition and a careful evocation procedure ensures the homogeneity of the (film) thickness obtained (see ref. 1 and references therein). During thermo-catalytic and thermo-photo-catalytic tests, the film was heated using a cartridge heater. The temperature of the layer was controlled and monitored by a temperature controller (Toho TTM-005) and a K-type thermocouple inserted into the reactor, contacting the surface of the catalyst. Minimal (below 1 ℃) axial temperature variation was reached with a cartridge heater (230 V; 500 W; "Resistencias RSI INCOLOID800") having controlled/compensated homogeneous heating. The UV irradiation was generated by four fluorescent UV lamps (Philips TL 6 W/08-F6T5 BLB, 6 W) symmetrically positioned outside the reactor. The lamps provide ca. 8 mW cm^{-2} intensity at the sample surface. The switching of the lamps only produces a modest change in temperature of less than 4 ℃. Full details about energy distribution and illumination intensity can be obtained from previous publications [1]. The reacting mixture used a CO_2:H_2 1:1 ratio in a nitrogen carrier (2/2/6 mL min^{-1}).

The catalytic properties were evaluated after ca. 4-5 h from the start of the irradiation, where a pseudo-stationary situation was reached. The concentrations of the reaction products were analyzed using an online mass spectrometer (Onmistart 300) and gas chromatograph (Agilent GC 7890) equipped with HP-PLOT-Q/HP-Innowax columns (0.5/0.32 mm I.D. × 30 m) as well as thermal conductivity and flame ionization detectors. Catalytic tests were repeated 3 to 4 times and error values were calculated from the corresponding set of data.

In this work we measure catalytic output with the help of three observables, the reaction rate, the quantum efficiency and the global energy balance of the reaction. The reaction rate (r) measures the number of hydrogen production molecules per surface area and time unit, but to analyze the thermo-photo production of hydrogen we define an “excess rate” (r_e) measured through Equation S1.

$$r_e = r_{\text{(Thermo-photo)}} - (r_{\text{(Photo)}} + r_{\text{(Thermo)}}) \qquad \text{(S1)}$$

Such “excess” rate measures the potential synergy occurring between both energy sources in the thermo-photo catalytic process. Synergy is thus measured as the excess (i.e. positive value) over the additive effect of light and heat in the reaction rate.

The second is the Quantum Efficiency (QE) parameter for hydrogen production. QE is defined by Equation S2 [2].

$$QE(\%) = 100 \times \frac{r\ (\text{mol m}^{-2}\text{s}^{-1})}{e^{a,s}\ (\text{Einstein m}^{-2}\text{s}^{-1})} \qquad \text{(S2)}$$

In this equation, r is the reaction rate and $e^{a,s}$ the average local superficial rate of photon absorption. Here, for the calculation of the quantum efficiency, we will use two different reaction rates, the normal one and the excess one. The use of the latter would allow to measure an “excess” quantum efficiency.

The rate of hydrogen production is measured using mass spectrometry and gas chromatography as previously outlined and normalized using the BET surface area of the sample. The local superficial rate of photon absorption ($e^{a,s}$) is defined by Equation S3. It follows from the equation corresponding to a pure photo-catalytic process but with an additional term that

accounts for the losses coming from charge emission with temperature [3]. In this equation, F_{As} is the fraction of light absorbed by the sample, q_{sup} the radiation flux at each position ($\underline{x} \equiv X_s$, Y_s, Z_s) of the catalytic film, and T_e is the thermal emission loss terminus.

$$e^{a,s}(\underline{x}) = \left(q_{sup}(\underline{x}) - T_e\right) F_{As} \quad \text{(S3)}$$

To obtain the radiation flow on the surface of the samples, we calculate first the impinging radiation flux from the lamps (q_n). Considering the coordinate system presented in Figure S1 and the geometry of the reactor (annular multi-lamp), the q_n can be determined by Equation S4 [1].

$$q_n\,(X_s,Y_s,Z_s) = \sum_{L=1}^{L=4} \sum_{\lambda} \int_{\varphi_{min,L(x,y)}}^{\varphi_{max,L(x,y)}} \int_{\Theta_{min(x,y,\varphi)}}^{\Theta_{max(x,y,\varphi)}} \frac{P_{\lambda,L}}{2\pi\, R_L\, Z_L} \sin^2\Theta \left(\left(\frac{X_s - X_L}{R}\right)\cos\varphi + \left(\frac{y_s}{R}\right)\sin\varphi\right) d\varphi\, d\Theta \quad \text{(S4)}$$

Where Xs, Ys, Zs are the coordinates of the points located on the surface of the catalytic film, and X_L, Y_L, Z_L which are the coordinates of the points located on the surface of the lamp. R type variables correspond to the radius of the cylinder supporting the sample (R) or of the lamp (R_L), see Figure S1. Angular variables (Θ,φ) are defined as described in Figure S1. Integration limits of Equation S4 are summarized in Equations S5-S12 and can be graphically visualized in Figure S1.

$$\varphi_1 = \tan^{-1}\left(\frac{X_L - Xs}{Y_L - Ys}\right) \quad \text{(S5)}$$

$$\varphi_2 = \sin^{-1}\left(\frac{R_L}{(X_L - X_s)^2 + (Y_L - Y_s)^2}\right) \quad \text{(S6)}$$

$$\varphi_{min} = \varphi_1 - \varphi_2 \quad \text{(S7)}$$

$$\varphi_{max} = \varphi_1 + \varphi_2 \quad \text{(S8)}$$

$$\Theta_{min}(\varphi) = \cos^{-1}\frac{-Z_s}{(X_{Lm}(\varphi) - X_s)^2 + (Y_{Lm}(\varphi) - Y_s)^2 + Z_s{}^2} \quad \text{(S9)}$$

$$\Theta_{max}(\varphi) = \cos^{-1}\frac{Z_L - Z_s}{(X_{Lm}(\varphi) - X_s)^2 + (Y_{Lm}(\varphi) - Y_s)^2 + Z_s{}^2} \quad \text{(S10)}$$

Where:

$$X_{Lm}(\varphi) = X_L + (X_s - Y_L)cos\,\varphi^2 + (Y_L - Y_s)(cos\,\varphi\, sin\,\varphi) - sin\,\varphi \sqrt{(R_L{}^2 - (X_s - X_L)\ cos\,\varphi + (Y_L - Y_s)\ sin\,\varphi\,)^2)}$$

(S11)

$$Y_{Lm}(\varphi) = Ys_i + (Y_L - Y_s)cos\,\varphi^2 + (X_s - X_L)(cos\,\varphi\, sin\,\varphi) - cos\,\varphi \sqrt{(R_L{}^2 - (X_s - X_L)\ cos\,\varphi + (Y_L - Y_s)\ sin\,\varphi\,)^2)}$$

(S12)

Finally, the q_{sup} x/y components (see Figure S1; Equation S13) can be determined using q_n and a radiation balance, which considers the main optical (Transmittance, F_i, and Reflectance, R_i) events occurring in all components of the reactor placed between the emission source and catalyst, i.e. glass and reaction media, as well on the catalytic film.

$$q_{sup}^{x,y} = f\ (\ q_n, F_i, R_i); i = \text{catalyst, glass, reaction media} \quad \text{(S13)}$$

A detailed description of the mathematical formulation to provide q_{sup} as a function of q_n (Equation S13) and the transmittance/reflectance optical measurements for each component of our reactor system can be found elsewhere [1,4]. As examples, the F_i parameters are displayed in Figure S2 for selected samples as a function of the illumination wavelength.

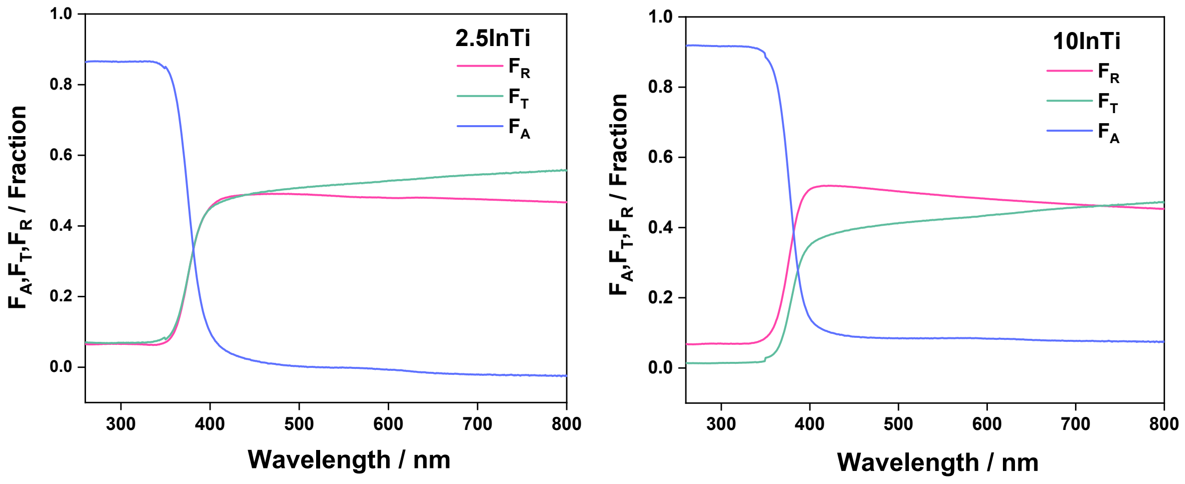

Figure S2. Fraction of light absorbed, transmitted or reflected by selected samples.

The T_e is a loss term and can be calculated using Equation S14. This Equation considers the emission of a body in a medium can be calculated using Plank´s law [3]. The radiation intensity per surface area unit is [3,5]:

$$T_e = \frac{n^2 \pi \, h \, T^4}{c^2} \int_0^{\infty} \frac{\gamma^3 \, \alpha(\gamma,T)}{e^{\frac{h\gamma}{k\,T}} - 1} \, d\gamma \qquad \text{(S14)}$$

Where γ is the photon frequency, h is the Plank´s constant, c is the speed of light, n is the refraction index of the solid, k is the Boltzmann´s constant, T is the temperature of the sample and α is the absorption efficiency that acts as an emissivity type factor as discussed in refs. 4,5. This T_e term is negligible at the temperatures of this work as it only makes a maximum correction of 4 parts per million to the local superficial rate of photon absorption values. This is at least 3-4 orders of magnitude below the standard error of the $e^{a,s}$ coefficient. Such a result is somehow expected as emission losses in titania-based (the dominant component) materials are known to occur at higher temperatures than here used [6].

Finally, an energy balance of the thermo-photo process is carried out to compare with the simple sum of the thermal and photo processes. Taking the equilibrium nature of the reaction [7], the denominator of equation 2 of the main text is obtained as:

$$r(CO) \times \Delta_f H^0_{CO} + r(H_2O) \times \Delta_f H^0_{H_2O} - r(H_2) \times \Delta_f H^0_{H2} - r(CO_2) \times \Delta f \, \Delta_f H^0_{CO_2} \quad (S15)$$

The $\Delta_f H^{\circ}$ for CO, H2O, H2 and CO2 are -110.6, -242, 0 and -393.8 kJ mol^{-1}, respectively. In this work, CO is obtained with nearly 100 % selectivity. Thus, the speed of formation of all molecules involved in the reaction is considered identical.

Catalytic and Characterization Results

Table S1. Comparison of activity parameters. The table includes literature reports considering the thermo-photo reverse water gas shift (RWGS) reaction. Results for samples with an asterisk consider activity parameters for the formation of CO but other products are detected.

Sample	Reaction Conditions	Rate (mmol g^{-1} h^{-1})<br>Excess Rate / %<br>Energy Efficiency / %	Reference
TiO_2 *	150 W Xe lamp<br>120 °C	0.4	8
TiO_2	500 W Xe lamp<br>250 °C	0.1	9
$InO_{3-x}(OH)_y$/SiNW	300 W Xe lamp<br>150 °C	0.22	10
1wt.% Pt/TiO_{2-x} *	150 W Xe lamp<br>120 °C	0.13	11
Ga-Cu/CeO_2	Solar Simulator<br>250 °C	0.83	7
InO_x/TiO_2	28 W UV lamps<br>250 °C	33<br>1.31	This work

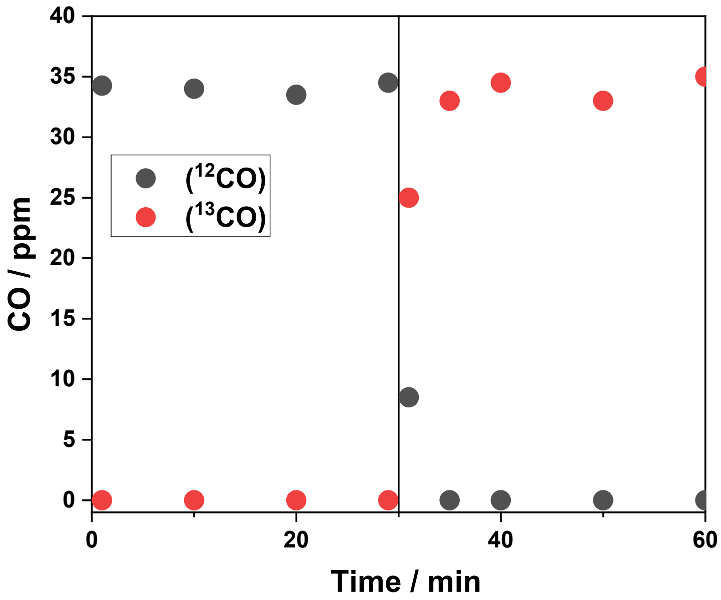

Figure S3. Isotopic switching experiment using $^{12}C/^{13}C$ marked CO_2. Thermo-photo conditions at 250 °C using the 10InTi sample. An arbitrary initial point for the time axis is presented.

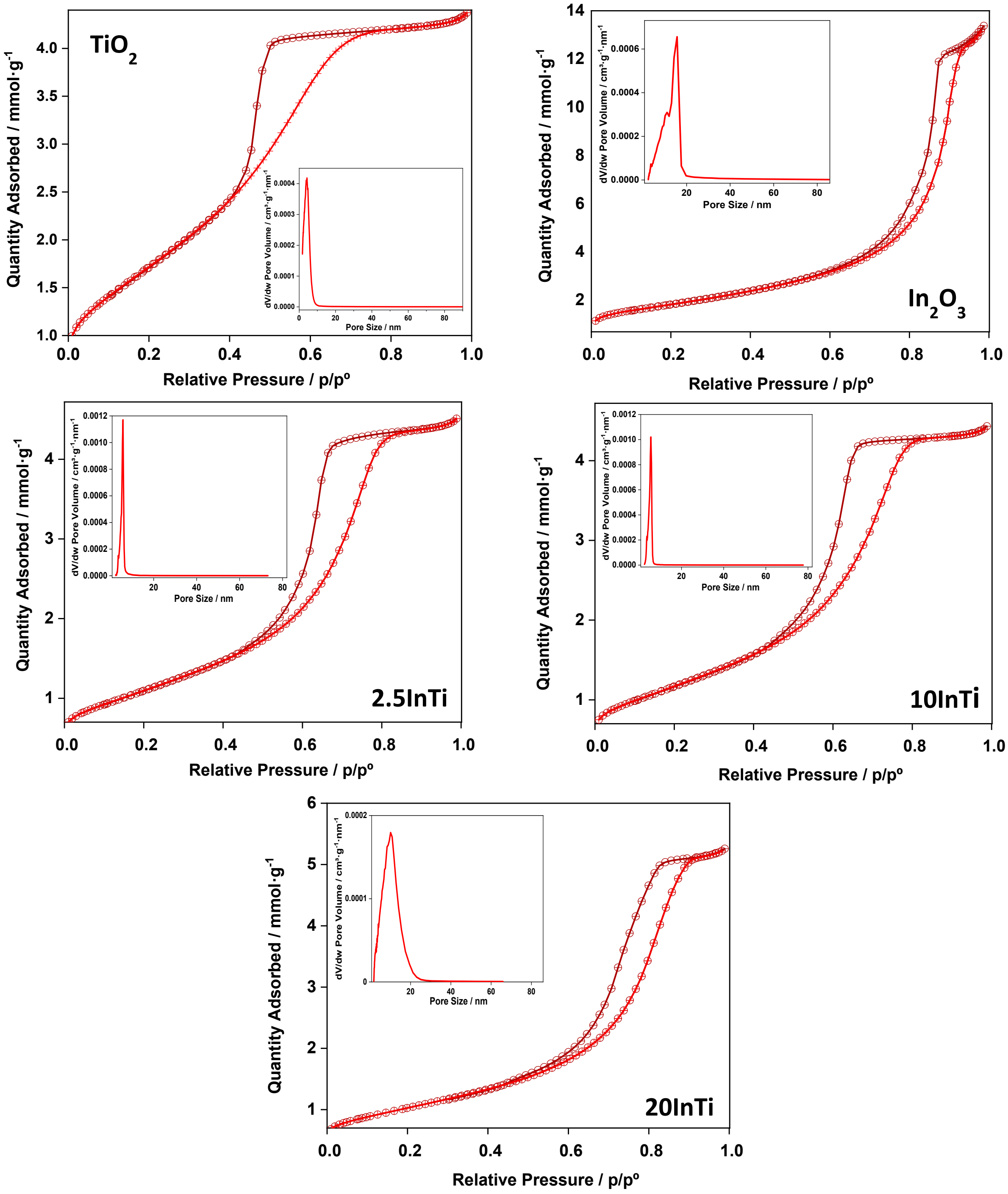

Figure S4. Nitrogen adsorption-desorption isotherms for selected samples. Insets display the pore size distribution of the corresponding sample.

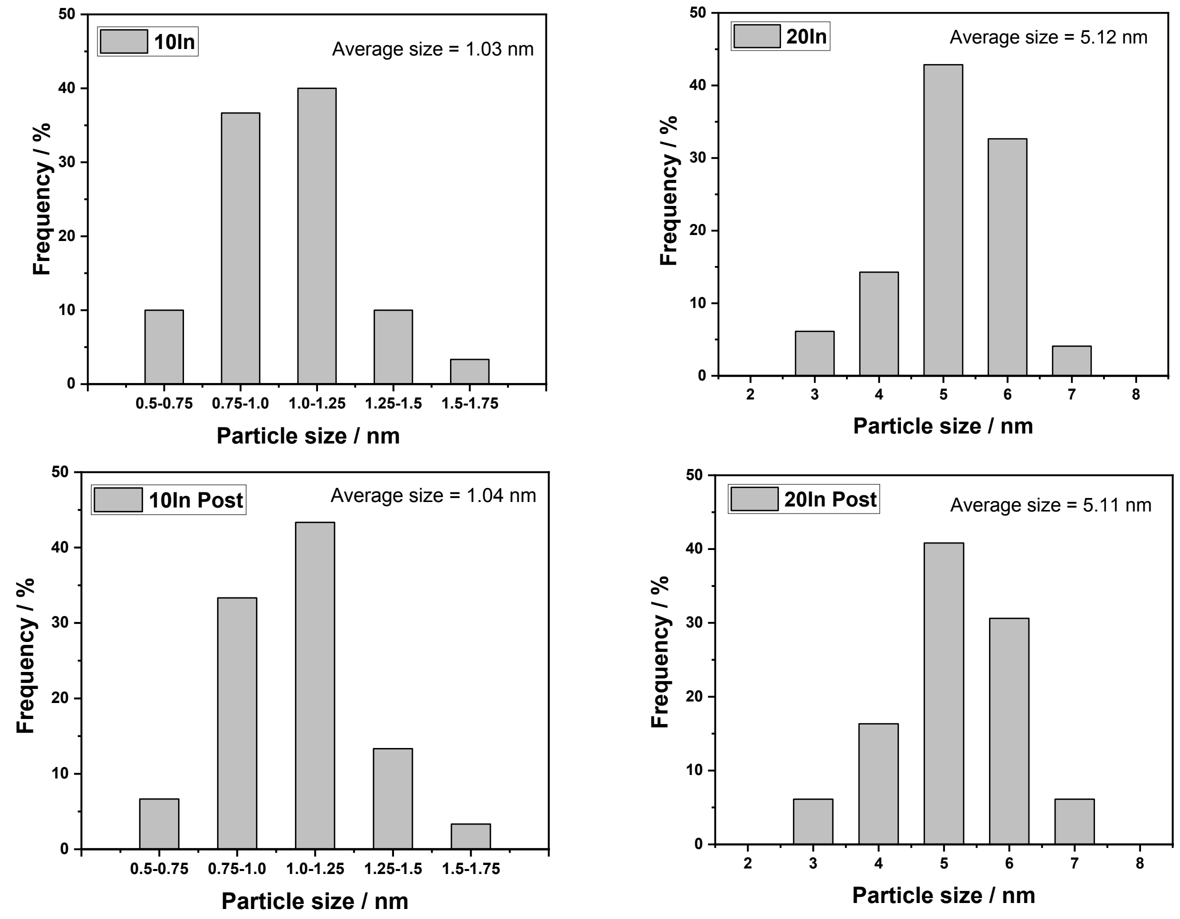

Figure S5. Primary particle size distributions for the In component for selected InTi samples. Pre- and post-reaction samples are presented.

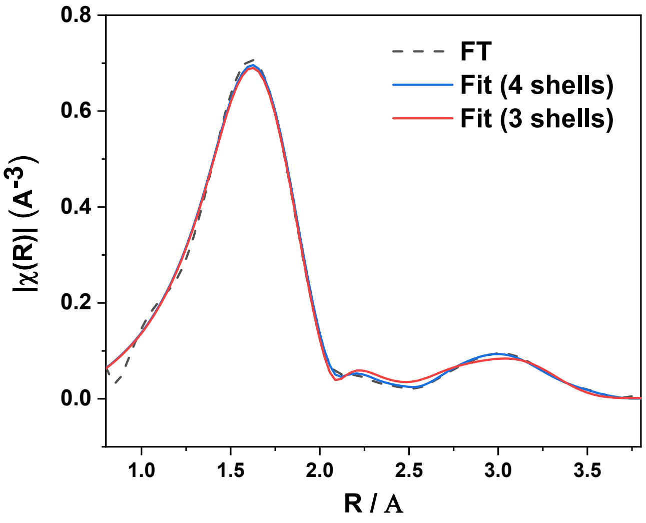

Figure S6. K^2-weighted Fourier Transform (FT) for experimental EXAFS data for the 10InTi sample and fitting results using models with 3 and 4 shells.

The statistical significance of the fitting outcome using a standard F-test of the variance is given by:

$$F = ((WRSS2-WRSS1)/(P2-P1)) / ((WRSS2/(n-P2)) \qquad (S16)$$

where WRSSi is the weighted sum of squared residuals of model i, n are the number of data points, and pi are the number of parameters corresponding to the three and four shells fittings; with $p2 > p1$. The F statistic will have an F distribution, with $((p2-p1)/2, (n-p2)/2)$ degrees of freedom. Under the null hypothesis, model number 2 (the one with 4 shells) does not provide a significantly better fit than model 1 (the one with 3 shells).

For our 10 InTi sample (data presented in Figure S6), the F-test takes the value of 4.057. This vale indicates that the 4-shell is statistically significant at a probability of 97.3 %. See main text for further details.

References

1 M.J. Muñoz-Batista, A. Kubacka, A.B. Hungría, M. Fernández-García. Heterogeneous photocatalysis: Light-matter interaction and chemical effects in quantum efficiency calculations. J. Catal. 330 (2015) 154–166. https://doi.org/10.1016/j.jcat.2015.06.021
2 S.E. Braslavsky, A.M. Braun, A.E. Cassano, A.V. Emeline, M.I. Litter, L. Palmisano, V.N. Parmon, N. Serpone. Glossary of terms used in photocatalysis and radiation catalysis (IUPAC Recommendations 2011). Pure Appl. Chem. 83 (2011) 931-1014. http://dx.doi.org/10.1351/PAC-REC-09-09-36
3 G.W. Kattawar, M. Eisner, Radiation from a homogeneous isothermal sphere. Appl. Optics 9 (1970) 2685-90.
4 G.E. Imoberdorf, H.A. Irazoqui, A.E. Cassano, O.M. Alfano. Photocatalytic Degradation of Tetrachloroethylene in Gas Phase on TiO2 Films: A Kinetic Study. Ind. Eng. Chem. Res. 44 (2005) 6075–6085. https://doi.org/10.1021/ie049185z
5 C.F. Bohm, D.R. Hoffman, Absorption and scattering of light by small particles. Whiley (1999) New York.
6 J. Xhen, P. Vincent, N.P. Blanchard, J. Nicolle, M. Choueib, V. Salles, A. Brioude. Physical properties of individual anatase TiO2 nanowires investigated by field emission in a transmission electron microscope. J. Vac. Sci. Technol. 30 (2012) 011801. https://doi.org/10.1116/1.3668121
7 B. Deng, H. Song, K. Peng, Q. Li, J. Ye, Metal-organic framework-derived Ga-Cu/CeO2 catalyst for highly efficient photothermal catalytic CO2 reduction, Appl. Catal. B Environ. 298 (2021) 120519. https://doi.org/10.1016/j.apcatb.2021.120519
8 Yu, F., Wang, C., Li, Y., Ma, H., Wang, R., Liu, Y., Suzuki, N., Terashima, C., Ohtani, B., Ochiai, T., et al. Enhanced solar photothermal catalysis over solution plasma activated TiO2. Adv Sci. , (2020) 2000204. 10.1002/advs.202000204
9 R. Sayago-Carro, M.N. Gómez-Cerezo, M. Fernández-García, A. Kubacka, Inverse-Type CoOx-TiO2 Catalysts for the Energy-Efficient Thermo-Photo Valorization of CO2. J. Clean. Prod. 2023, 429, 139542. https://doi.org/10.1016/j.jclepro.2023.139542
10 L.B. Hoch, P.G. O'Brien, A. Jelle, A. Sandhel, D.D. Perovic, C.A. Mims, G.A. Ozin, Nanostructured indium oxide coated silicon nanowire arrays: a hybrid photothermal/photochemical approach to solar fuels. ACS Nano 10 (2016) 9017-9025. 10.1021/acsnano.6b05416.

11 F. Yu, C. Wang, H. Ma, M. Song, D. Li, Y. Li, S. Li, X. Zhang, Y. Liu, Revisiting Pt/TiO2 photocatalysts for thermally assisted photocatalytic reduction of CO2. Nanoscale 12 (2020) 7000-7010. 10.1039/c9nr09743k
